# Nodule-forming pseudoangiomatous stromal hyperplasia of the breast: case report

**DOI:** 10.11604/pamj.2019.33.180.17832

**Published:** 2019-07-08

**Authors:** Faten Limaiem, Saâdia Bouraoui

**Affiliations:** 1 Department of Pathology, Mongi Slim Hospital, La Marsa, Tunisia; 2 Tunis Faculty of Medicine, University of Tunis El Manar, El Manar, 1007, Tunisia

**Keywords:** Breast, pseudoangiomatous stromal hyperplasia, pathology, immunohistochemistry

## Image in medicine

Pseudoangiomatous stromal hyperplasia (PASH) is an uncommon benign proliferation of fibrous stroma, containing slitlike pseudovascular spaces lined by myofibroblasts. Less than 200 cases of PASH have been described in the English literature with the largest series including 40 cases. A 48-year-old woman with no particular past medical history, presented with a complaint of a slow, progressive, painless left breast mass. The patient underwent mammography which revealed a well delineated left breast nodule with microcalcifications classified BI-RADS 4. An excisional biopsy was performed on the mass to confirm the histological diagnosis. The specimen was a well-circumscribed whitish tumor measuring 3 x 3 cm with several microcysts (A). Histological examination revealed proliferation of fibrous stroma, with spindle cells and a network of slit-like empty clefts within acellular hyalinized stroma (B). The spindle cells were positive for CD34 and smooth muscle actin but were negative for Factor VIII. The final pathological diagnosis was nodular PASH. Postoperative course was uneventful and the patient is still being followed-up.

**Figure 1:**
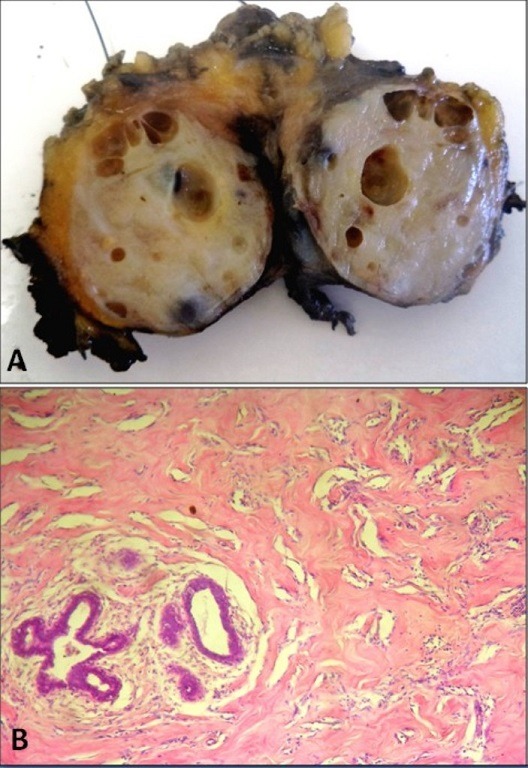
(A) gross appearance of nodular PASH. The lesion is circumscribed with a whitish cut surface and several microcyts ranging in size from 1 to 6 mm; (B) numerous slit-like spaces are present in dense collagenous breast stroma. The slitlike spaces are lined by benign spindle cells. No cytologic atypia is present. (Hematoxylin and eosin, magnification × 400)

